# Current clinical and translational challenges in temporomandibular joint reconstruction

**DOI:** 10.3389/fbioe.2025.1590021

**Published:** 2025-09-18

**Authors:** Helena Baecher, Bhagvat Maheta, Lisa-Marie Lottner, Adriana C. Panayi, Samuel Knoedler, Fernando Guastaldi, João F. Mano, Steffen Koerdt, Carsten Rendenbach, Max Heiland, Leonard Knoedler, Christian Doll

**Affiliations:** ^1^ Department of Oral and Maxillofacial Surgery, Charité – Universitätsmedizin Berlin, Corporate Member of Freie Universität Berlin and Humboldt-Universität zu Berlin, Berlin, Germany; ^2^ California Northstate University College of Medicine, Elk Grove, CA, United States; ^3^ Department of Oral and Maxillofacial Surgery, University Hospital Regensburg, Regensburg, Germany; ^4^ Department of Plastic Surgery and Hand Surgery, Klinikum rechts der Isar, Technical University of Munich, Munich, Germany; ^5^ Department of Oral and Maxillofacial Surgery, Massachusetts General Hospital, Harvard School of Dental Medicine, Boston, MA, United States; ^6^ Department of Chemistry, CICECO – Aveiro Institute of Materials, University of Aveiro, Aveiro, Portugal

**Keywords:** temporomandibular joint reconstruction, TMJ replacement, tissue engineering, bioprinting, 3D printing, computer-aided design, computer-aided manufacturing, CAD/CAM

## Abstract

Total joint reconstruction (TJR) is essential for the management of end-stage temporomandibular joint (TMJ) disorders. Current reconstruction techniques include the use of autologous grafts, such as chondrocostal tissue or fibula, and alloplastic TMJ replacement systems. Commercially available TMJ replacement systems provide both stock and customized prostheses. Advances in computer-aided design/computer-aided manufacturing technology, three-dimensional printing, and virtual surgical planning have accelerated the trend toward individualized TMJ prostheses, enhancing anatomical adaptation, intraoperative efficiency, and postoperative outcomes. A promising alternative under preclinical investigation is TMJ tissue engineering, a regenerative approach utilizing scaffolds, stem cells, and growth factors to reconstruct specific TMJ components, including the skeletal condyle, fibrocartilaginous disc, and glenoid fossa. Bioprinting has further transformed the field by enabling the creation of complex, multi-tissue structures with cellular viability and functionality. Techniques such as integrated tissue and organ printing and volumetric printing have shown promise for enhancing graft performance by improving scaffold heterogeneity. However, these advanced approaches remain in the preclinical stage and require critical evaluation before clinical translation. Despite these advancements, challenges such as high costs, technical complexities, and the need for extensive, robust datasets persist. Continued research into novel biomaterials, advanced biofabrication techniques, and digital surgical technologies, supported by larger preclinical and *in vitro* studies, is imperative to address these limitations and advance clinical applicability.

## Introduction

Temporomandibular disorders (TMD) affect approximately 31%–34% of the global population, with a higher prevalence among females compared to males (Zieliński et al., 2024; Valesan et al., 2021). As a significant public health concern, the true burden of TMD is likely underestimated due to undiagnosed cases (Spotts, 2017). Although robust data on the proportion of TMD patients undergoing surgery are scarce, approximately 6% receive surgical intervention (Salinas et al., 2022). In severe instances, including end-stage temporomandibular joint (TMJ) diseases, congenital abnormalities, neoplasms, or trauma, surgical reconstruction of the TMJ may become necessary (Resnick, 2018; He et al., 2011; Schmidt et al., 2022; Gonzalez-Perez et al., 2023). In 2014, 572 alloplastic TMJ prostheses were implanted in the United States; projections indicate that 902 procedures are expected by 2030 (Onoriobe et al., 2016). However, techniques for TMJ reconstruction represent an essential area of research that has undergone constant development over the last few years.

**FIGURE 1:**
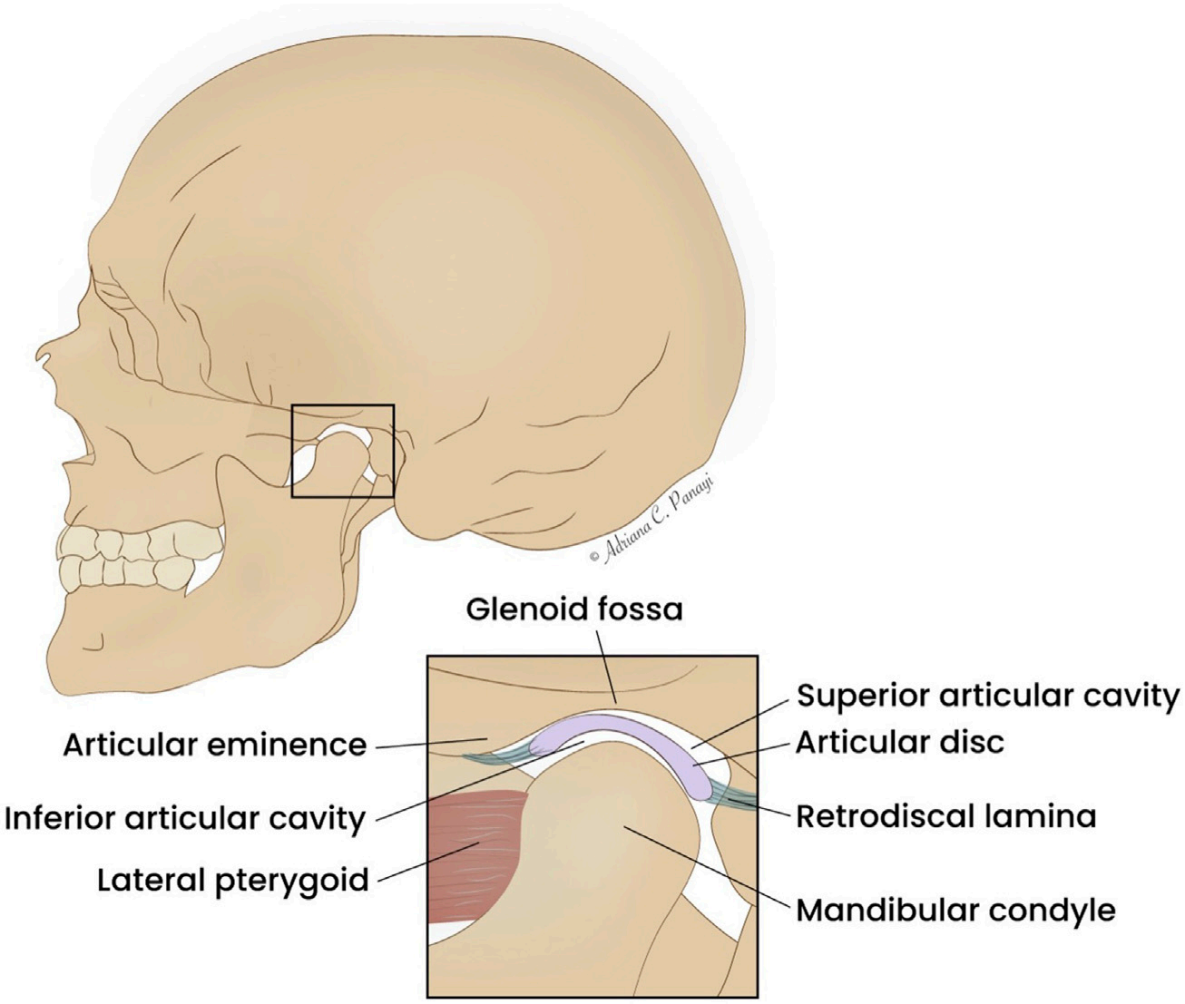
Schematic illustration of the TMJ. Created with Adobe Illustrator (Version 29.6, Adobe Inc., San Jose, California, United States).

The surgical approach varies based on the extent of pathological involvement, ranging from arthroscopic techniques or targeted discectomy to resection of the mandibular condyle, or total resection of the TMJ (Breik et al., 2016; Ângelo et al., 2022; Kreutziger, 1994). A schematic illustration of the TMJ is provided in Figure 1.

Modern strategies for TMJ reconstruction integrate diverse surgical techniques, including revascularized tissue transfers, costochondral grafting, and alloplastic TMJ replacement. The choice of procedure depends on several patient-specific factors, such as age, underlying pathology, skeletal maturity, and the risk of compromising the contralateral joint’s function (Imola and Liddell, 2016). In most clinical scenarios, total TMJ replacement using alloplastic prostheses is the preferred approach. In contrast, autologous graft reconstruction is commonly chosen for growing children, patients undergoing adjuvant radiation following malignant tumor removal, and individuals in low-income settings (Pedersen et al., 2021; Borbon et al., 2023). The traditional view supports the use of autologous grafts in skeletally immature patients, emphasizing their potential to grow with the child and thereby reduce the risk of future facial asymmetry (Ko et al., 1999; Awal et al., 2018). Recent studies, however, have demonstrated the applications of customized alloplastic TMJ prostheses even in skeletally immature patients (Alba et al., 2024; Sinn et al., 2021; Hashemi et al., 2024).

Over recent decades, the development and application of alloplastic materials, along with advances in tissue-engineered solutions, have significantly expanded the options for TMJ replacement (Salash et al., 2016).

In the era of computer-aided design and manufacturing (CAD/CAM), three-dimensional (3D) printing, and virtual surgical planning (VSP), TMJ reconstruction has undergone significant advancements.

As a result, patient-specific TMJ prostheses designed through VSP have become a widely established approach, continuously undergoing refinements. Future perspectives focus on tissue engineering and bioprinting of TMJ tissues, offering the potential for biomimetic innovations. However, these technologies remain in preclinical stages and should be critically evaluated.

Furthermore, initial efforts have been made to integrate augmented reality and virtual reality into the field of TMJ surgery. These technologies provide preoperative support for condylar resection planning, prosthesis selection, and the identification of potential interferences. Additionally, they offer intraoperative real-time guidance for osteotomies, prosthesis placement, screw fixation, and occlusion verification (Niloy et al., 2024).

Despite these advancements, there remains a scarcity of comprehensive studies that condense the latest innovations in TMJ surgery. This research gap leaves untapped potential to leverage recent findings to improve TMJ patient care. To fill this research gap, this review aims to provide an encompassing overview of the recent developments in TMJ reconstruction and distill the most promising research findings.

## State of the art–established techniques in TMJ reconstruction

### Virtual surgical planning

Early TMJ surgery relied on manually shaping autologous grafts, such as costochondral or fibula free flaps, guided by anatomical landmarks and refined through intraoperative adjustments (Wax et al., 2000; Munro and Chir, 1980). However, this freehand shaping of the bone grafts into an accurately fitting condyle head is challenging, and inaccuracies in contouring the new TMJ can lead to mandibular dysfunction, impacting swallowing, speaking, and chewing (Wax et al., 2000). To improve outcomes, VSP integrated digital simulations and 3D models into preoperative workflows, enhancing precision (Raccampo et al., 2023). Advances in 3D printing and CAD/CAM technologies now enable tools like 3D-printed drilling guides, placement guides and patient-specific osteosynthesis plates in maxillofacial surgery, which aid in accurate graft shaping and synthesis (Myers et al., 2021; Rodby et al., 2014; Hadad et al., 2023).

**FIGURE 2:**
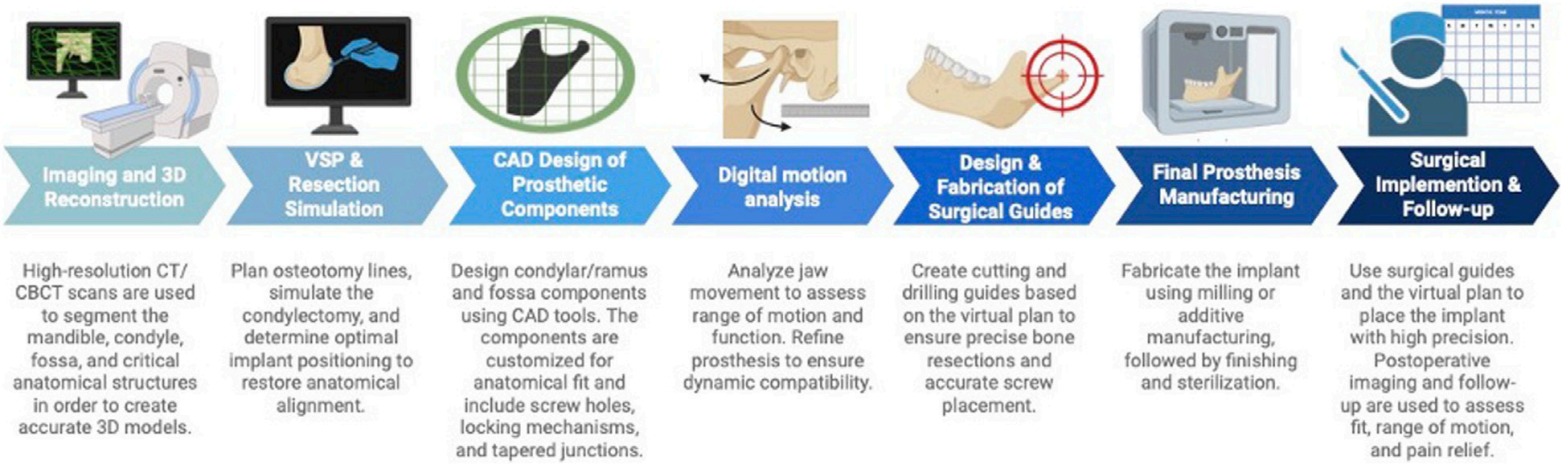
Workflow of VSP and CAD/CAM-based fabrication of TMJ prostheses. Created with BioRender (BioRender Inc., Toronto, Canada). Abbreviations: CAD/CAM. computer-aided design/computer-aided manufacturing, VSP: virtual surgical planning.

In current clinical workflows, high-resolution computed tomography (CT) or cone-beam computed tomography (CBCT) scans are used to capture bony anatomy, in select cases supplemented by magnetic resonance imaging (MRI) for visualization of soft tissues and vascular structures. These datasets are imported as Digital Imaging and Communications in Medicine (DICOM) files into dedicated surgical planning software (e.g., Mimics, Materialise NV, Leuven, Belgium), where they are co-registered within a unified coordinate system to ensure accurate anatomical alignment. Using semi-automated or AI-assisted segmentation tools, individual anatomical regions, particularly the TMJ, are isolated and converted into 3D surface files (e.g., STL format). In subsequent design platforms (e.g., 3-matic, Materialise NV, Leuven, Belgium), osteotomy lines are defined, either as simple planar cuts or complex freeform curves, based on the clinical scenario. Prosthetic components are virtually designed, often in collaboration between biomedical engineers and surgeons, and adjusted according to anatomical landmarks and resection planes. Surgical guides for osteotomies and drilling are then modeled within the same planning environment and manufactured via 3D printing using biocompatible materials. These guides are typically aligned with the planned implant trajectory to ensure congruent intraoperative placement. Additionally, anatomical models of the mandible and prosthetic replicas may be printed to assist with surgical rehearsal and intraoperative reference. All components are thoroughly sterilized and quality-controlled prior to clinical use, enabling accurate, reproducible execution of the preoperative plan (Raccampo et al., 2023). A workflow of VSP and CAD/CAM-based fabrication of TMJ prostheses is represented in Figure 2.

The integration of VSP with personalized CAD/CAM-produced devices was initially designed to enhance efficiency and support surgeons in TMJ reconstruction. This approach offers i) reduced surgical time, ii) improved accuracy through cutting and placement guides and precalculated contact surfaces, iii) enhanced aesthetic results iv) visualization of patient-specific anatomical variations before and during surgery, and v) precise margins in tumor resection scenarios prior to TMJ reconstruction (del Castillo Pardo de Vera et al., 2024; Mian et al., 2021; Dowgierd et al., 2021). With regard to alloplastic TMJ replacement, improved accuracy of the condylar position, reduced screw stress, and diminished contact stress on the condylar component remain the most significant benefits (Mian et al., 2021). Recent studies on the accuracy of TMJ prostheses implemented with VSP report highly precise positioning associated with long-term surgical success in the form of enhanced osseointegration and reduced micromovements (Neuhaus et al., 2021).

A study by Sawh-Martinez et al. evaluated the position of the condyle following mandibular reconstruction using CT imaging. The comparison between mandibular reconstructions with and without VSP demonstrated that VSP significantly improved precision, particularly by reducing superior and lateral shifts of the ipsilateral condyle, as well as minimizing changes in the condylar and condylar neck angles (Sawh-Martinez et al., 2017).

### Current TMJ alloplastic replacement systems

Several commercially available TMJ replacement systems exist, including the Zimmer Biomet Microfixation System (Jacksonville, United States), patient-specific TMJ Concepts by Stryker (Ventura, United States), Materialise’s custom-made TMJ Total Arthroplasty System (Leuven, Belgium), and IPS Implants® by KLS Martin (Tuttlingen, Germany).

The Zimmer Biomet system offers both stock and customized prostheses, while the TMJ replacement systems by TMJ Concepts, Materialise, and KLS Martin are exclusively patient-specific (Kanatsios et al., 2018; Jones, 2011; Materialise, 2023; KLS Martin Group, 2024). Stock prostheses provide advantages such as immediate availability and lower costs compared to customized systems. Furthermore, preoperative planning is less time-intensive for stock prostheses (Gonzalez-Perez et al., 2016). However, these advantages are offset by the need for intraoperative adaptation, which may prolong surgical procedures (Olate et al., 2023). A significant limitation of stock prostheses is their reduced precision, as they cannot be perfectly tailored to individual patients. For instance, Abramowicz et al. reported that 23% of Biomet Microfixation stock prostheses failed to fit stereolithographic models of patients who subsequently required patient-specific joint prostheses (Abramowicz et al., 2012).

On the other hand, customized prostheses offered by all the mentioned manufacturers provide the benefits of patient-specific designs, enabling simplified intraoperative workflows, reduced bone modification, and enhanced precision in fitting (Mercuri, 2013). However, these advantages come at a higher cost, largely due to their complex manufacturing processes (Zheng et al., 2019).

All four systems are two-component designs consisting of a mandibular condyle and a glenoid fossa. The mandibular components of the Zimmer Biomet (stock/customized), TMJ Concepts, and Materialise systems comprise a cobalt-chromium-molybdenum (Co-Cr-Mo) alloy condylar head and a titanium alloy body, which is either entirely composed of titanium or features a plasma-sprayed titanium coating (Materialise, 2023; KLS Martin Group, 2024; Giannakopoulos et al., 2012; Aagaard and Thygesen, 2014). In contrast, the mandibular component of the KLS Martin system is manufactured entirely from a titanium alloy (KLS Martin Group, 2024). Remarkably, Zimmer Biomet offers a fully titanium alternative for the mandibular component in patients with verified nickel or Co-Cr allergies (Granquist et al., 2018). For all five systems, the fossa components are fabricated from ultra-high molecular weight polyethylene (UHMWPE). In the Zimmer Biomet system (stock/customized), the entire fossa component is composed of UHMWPE, whereas in the other systems, the UHMWPE articulating surface is secured to a titanium mesh backing (TMJ Concepts) or a metal base (Materialise and KLS Martin) (Materialise, 2023; KLS Martin Group, 2024; Giannakopoulos et al., 2012; Aagaard and Thygesen, 2014). Notably, all five systems provide cutting and/or positioning guides (Materialise, 2023; KLS Martin Group, 2024; Brown et al., 2016; Sembronio et al., 2021).

The stock Zimmer Biomet System received full approval by the US Food and Drug Administration (FDA) in 2005 and represents a well-established option in TMJ reconstruction, with available long-term outcomes (Giannakopoulos et al., 2012).

In contrast, the systems by Materialise and KLS Martin are relatively recent advancements. Consequently, long-term studies and robust evidence-based data for these systems are currently lacking. Notably, there exists a retrospective study on customized TMJ prostheses by Engimplan/Materialise Company (Rio Claro, Brazil), evaluating 22 patients over 2 years (Leandro et al., 2022). However, the high level of personalization offered by these prostheses, combined with insertion aids such as drilling and osteotomy guides, surgical splints, and TMJ trial implants for precise fossa component placement, provides significant advantages (Materialise, 2023; KLS Martin Group, 2024).

A summary of the materials used in alloplastic TMJ replacement systems is provided in Table 1.

**TABLE 1:** Summary of materials used in different alloplastic TMJ replacement systems.

Manufacturer		Zimmer Biomet	Stryker	Materialise	KLS Martin
Type		Customized/stock	Customized	Customized	Customized
Mandibular component	Condylar head	Co-Cr-Mo/Ti-6Al-4V (for patients with verified nickel or Co-Cr allergy)	Co-Cr-Mo	Co-Cr	Ti-6Al-4V
	Ramus	Plasma-sprayed titanium alloy coating/Ti-6Al-4V (for patients with verified nickel or Co-Cr allergy)	Ti-6Al-4V	Ti-6Al-4V	Ti-6Al-4V
Fossa component		UHMWPE	UHMWPE articulating surface on a titanium mesh backing	UHMWPE articulating surface on a titanium alloy base plate	UHMWPE articulating surface on a metal base
Screws		Ti-6Al-4V	titanium alloy	titanium alloy	Ti-6Al-4V
Surgical aids		Drilling and osteotomy guides (UHMWPE/stereolithography resin)	Drilling and osteotomy guides	Drilling and osteotomy guides (titanium/polyamide), surgical splint, tmj trial implants	Drilling and osteotomy guides with integrated steel sleeves

Abbreviations: TMJ: temporomandibular joint, Co-Cr-Mo: Cobalt-Chromium-Molybdenum alloy, Ti-6Al-4V: Titanium-Aluminium-Vanadium alloy, UHMWPE: ultra-high molecular weight polyethylene.

### Alloplastic vs. autologous reconstruction

Before the advent of TMJ alloplastic replacement systems, and in cases where such implants are contraindicated, autologous TMJ reconstruction was, and continues to be, the preferred method. The most commonly used grafting tissues include costochondral grafts, sternoclavicular grafts, and free fibula grafts (Sidebottom, 2013).

Complications associated with costochondral grafts are reported in 51%–58% of patients and include infection, facial nerve weakness, malocclusion, and graft fracture due to overload. Especially, (re)ankylosis and graft overgrowth represent common issues (Awal et al., 2018; Sidebottom, 2013; Saeed et al., 2003). Revision surgery or implant removal is required in approximately 42% of cases (Saeed et al., 2003). Significant drawbacks of costochondral grafts are the unpredictable growth and the donor site morbidity, which can include chronic costochondritis pain and the risk of pleural disruption (Sidebottom, 2013; Sekhoto et al., 2019).

TMJ reconstructions using sternoclavicular grafts have been associated with complications such as joint ankylosis, foreign body giant cell reaction, graft destruction or fracture, and donor site morbidity, including clavicle fractures. Graft survival rates were 50% in patients with inflammatory TMJ pathology but reached 93% in those without inflammatory conditions or prior alloplastic TMJ reconstruction (Wolford et al., 1994).

Reconstruction of the mandibular condyle using a fibula free flap is predominantly performed in cases of mandibulectomy involving the condyle. Wax et al. reported postoperative fibular head displacement out of the fossa in 12% of cases, hypoperfusion of the graft requiring a second venous anastomosis in 6%, and asymptomatic bone exposure in 6% (Wax et al., 2000). In further studies, graft survival was reported as 100%. Preservation of the articular disc in most patients likely contributed to the absence of TMJ ankylosis (Wax et al., 2000; Gravvanis et al., 2017; Powers et al., 2022). Donor site morbidity is described in the literature as relatively low, including a limited range of motion in the ankle, ankle instability, and gait abnormalities (Rendenbach et al., 2016).

In clinical practice, alloplastic TMJ prostheses are the primary choice for TMJ replacement or reconstruction. The literature indicates low rates of removal and revision for these devices. Amarista et al. reported revision rates of 3% for patient-specific TMJ Concepts prostheses, 3% for stock Zimmer Biomet prostheses, and 2% for custom Zimmer Biomet prostheses (Amarista et al., 2020). Even in cases of extended TMJ replacement, treatment with TMJ prostheses has been demonstrated to be a safe and effective option for patients with TMJ deficits involving the mandible and/or the zygomatic arch (Gerbino et al., 2024).

Adverse events associated with alloplastic TMJ replacements include infection, nerve injuries (i.e., facial nerve, trigeminal nerve), neuropathic pain, and hearing disturbances, with an overall incidence of 5%–19% (Aagaard and Thygesen, 2014; Neff et al., 2020). A meta-analysis by Lima et al. identified the most common complications as facial nerve paresis or paralysis (8%), sensory disturbances (2%), heterotopic bone formation (1%), and infections (1%) (Peres Lima et al., 2023). Functional complications, such as hardware loosening and ankylosis, have also been reported, while multiple surgeries prior to TMJ replacement are associated with poorer outcomes (Neff et al., 2020; Rajkumar and Sidebottom, 2022; Sanovich et al., 2014; Handa et al., 2023). Notably, hypersensitivity or allergic reactions to prosthetic materials occur in approximately 11% of patients (Neff et al., 2020).

Implant survival rates are high, with a 2024 meta-analysis reporting a 97% survival rate over follow-up periods ranging from 12 months to 21 years (Lima et al., 2024). A long-term study by Wolford et al. documented a 100% implant survival with a median follow-up of 21 years (Wolford et al., 2015). A comparison of different options for TMJ reconstruction is provided in Table 2.

**TABLE 2:** Comparison of the different options for TMJ reconstruction.

	Autologous	Alloplastic	Tissue-engineered
Materials used	Costochondral grafts Sternoclavicular grafts Free fibula grafts	Co-Cr-Mo Ti-6Al-4V UHMWPE	HA, PLA, PCL, PGA, PLLA, PLGA, PEGDA, calcium phosphate, ECM sheets, fibrin, collagen, chitosan, alginate living cells additives
Advantages	Reduced costs Traditional solution for immature patients	No donor site morbidity, unlimited availability, reduced operation time	No donor site morbidity, unlimited availability, reduced operation time, biologic alternative
Disadvantages	Donor site morbidity, increased operation time, unpredictable growth	Increased costs, no growth, hypersensitivity	Elaborate and costly production, not clinically available
Success Rates	50%–93% (Awal et al., 2018; Sidebottom, 2013; Saeed et al., 2003; Wolford et al., 1994)	95%–97% (Amarista et al., 2020; Lima et al., 2024)	-

Abbreviations: TMJ: temporomandibular joint, Co-Cr-Mo: Cobalt-Chromium-Molybdenum alloy, Ti-6Al-4V: Titanium-Aluminium-Vanadium alloy, UHMWPE: ultra-high molecular weight polyethylene, ECM: extracellular matrix, PCL: polycaprolactone, PLA: polylactide, PGA: polyglycolic acid, PLLA: poly-L-lactic acid, PLGA: poly-L-lactic-co-glycolic acid, PEGDA: photopolymerized hydrogel polyethylene glycol diacrylate, HA: hydroxylapatite.

### TMJ replacement in children

Reconstruction of the TMJ in children presents a unique clinical challenge. The most common underlying conditions necessitating TMJ reconstruction include congenital deformities, neoplasms, ankylosis, and progressive resorptive diseases (Resnick, 2018). Early reconstruction of the mandible and TMJ has been shown to reduce the risk of facial asymmetry and growth disturbances affecting the mandible, maxilla, and midfacial skeleton. In addition, early intervention may mitigate the development of malocclusion, symptoms of CMD, obstructive sleep apnea, and psychosocial issues (Resnick, 2018). Despite early intervention, more than 50% of pediatric patients exhibit recurrent facial asymmetry by the time they reach skeletal maturity (Pluijmers et al., 2014). Traditional surgical paradigms advocate the use of autologous grafts in skeletally immature patients due to their potential for growth and remodeling, theoretically reducing the likelihood of future asymmetry (Ko et al., 1999; Awal et al., 2018). Costochondral grafts and vascularized free fibula flaps are the most established autologous options in this population. However, costochondral grafts are primarily composed of cortical bone and rely heavily on surrounding tissues for revascularization, which increases the risk of postoperative resorption compared to vascularized flaps (Resnick, 2018). Furthermore, the growth behavior of costochondral grafts is unpredictable. Reports have documented cases of overgrowth, undergrowth, and even lack of growth (Awal et al., 2018). Recent studies have explored the use of customized alloplastic TMJ prostheses in skeletally immature patients (Yang et al., 2015). Although alloplastic TMJ prostheses are considered the treatment of choice in adults, their use in growing patients remains controversial due to concerns about interference with craniofacial development and the potential need for future revision surgeries (Alba et al., 2024; Sinn et al., 2021; Hashemi et al., 2024). However, many children requiring TMJ reconstruction already exhibit severely disrupted mandibular growth patterns, and over half of autologous reconstructions eventually necessitate surgical revision (Keyser et al., 2020). A 10-year follow-up study of pediatric costochondral grafts reported revision surgery in 33% of cases due to recurrent ankylosis, in 16% due to overgrowth, and in 7% due to undergrowth (Awal et al., 2018).

A recent survey of 14 pediatric patients treated with customized alloplastic TMJ prostheses reported only two postoperative complications requiring surgical intervention: one infection and one case of heterotopic ossification. Bilateral reconstruction was performed in nine cases, and unilateral reconstruction in five. All patients demonstrated improved mandibular function, increased maximum interincisal opening (MIO), and no evidence of asymmetrical growth (Keyser et al., 2020). In a case series by Sinn et al., five pediatric TMJ reconstructions were performed using both stock and customized alloplastic devices. All cases showed improved MIO. Secondary procedures included orthodontic treatment and the use of a palatal expander in one patient, and surgical scar revision in another. Collectively, while the use of customized alloplastic TMJ prostheses in children remains controversial, emerging evidence suggests they may be a viable alternative in selected cases, particularly after failed autologous reconstruction.

## Novel innovations and future perspectives

### Total joint replacement

Total joint replacement is required in cases of end-stage TMJ disorders and involves the surgical replacement of the TMJ with an alloplastic prosthesis (Neff et al., 2020). The aim of total TMJ replacement is to restore both morphology and function, striving to replicate the native TMJ as closely as possible (Yadav et al., 2021). The FDA-approved materials for alloplastic joint prostheses include cobalt–chromium alloys, commercially pure titanium (cpTi), alloyed titanium (Ti6Al4V), and UHMWPE (Yadav et al., 2021).

Research is increasingly focused on optimizing accurate fitting by customized joint prostheses to restore optimal functions. A study by Ingawale et al. introduced a patient-specific TMJ prosthesis with novel features designed to enhance adaptation to each patient’s anatomic conditions. The surgeon can customize the shape and center of rotation of the condylar head, with the design being precisely adapted to the load-bearing conditions. The prosthesis incorporates perforated notches that extend into the host bone at implantation. The glenoid fossa design facilitates sufficient rotation and allows for anterior-posterior and medio-lateral translation. A modified design, featuring a rectangular slot with curved anterior and posterior edges and circumferential stabilization for the condylar head, ensures accurate fitting on the skeletal surface. Consequently, this study demonstrates that the introduced approach holds promise for stable total TMJ reconstruction, validated under both functional/normal and para-functional/worst-case TMJ loading scenarios (Ingawale and Goswami, 2022).

De Meurechy et al. developed an innovative, patient-specific 3D-printed titanium alloy TMJ replacement system designed to restore laterotrusive movements through the reinsertion of the lateral pterygoid muscle. Utilizing a sheep model, the study evaluated the efficacy of a HadSat® diamond-like carbon (H-DLC) coating for the condylar head, combined with a machined UHMWPE fossa component enriched with Vitamin E and subjected to γ-irradiation. The findings advocate for the combined use of H-DLC-coated titanium for the condyle and Vitamin E-stabilized UHMWPE for the fossa as a superior option for TMJ implant systems (De Meurechy et al., 2022).

In a sheep model, another research group investigated a novel, customized TMJ prosthesis that permits the reinsertion and integration of the lateral pterygoid muscle (LPM) into the prosthesis. This approach aimed to preserve LPM function and reduce excessive load on the contralateral joint through optimized load distribution. The methodology involved preserving the LPM enthesis and reattaching it to a scaffold at the condylar neck of the TMJ prosthesis. Again, an H-DLC coating was applied to the condylar head. Histological analysis demonstrated a functional fibrotic reintegration of the LPM onto the prosthesis, thereby successfully restoring muscle function (Mommaerts et al., 2025).

### Tissue engineering–the future of TMJ reconstruction?

Tissue engineering for TMJ reconstruction focuses on three components: i) the skeletal condyle, ii) the fibrocartilaginous disc, and iii) the glenoid fossa and articular eminence (Aryaei et al., 2016; Acri et al., 2019). This process combines scaffolds, cells, and growth factors, but optimizing these elements for each anatomical component of the TMJ remains challenging (Acri et al., 2019).

Biological scaffolds which can be used for the reconstruction of the articular disc include decellularized extracellular matrix (ECM) sheets, fibrin, collagen, and chitosan (Acri et al., 2019). Synthetic scaffolds include PLA, PCL, polyglycolic acid (PGA), poly-L-lactic acid (PLLA), poly-L-lactic-co-glycolic acid (PLGA), and photopolymerized hydrogel polyethylene glycol diacrylate (PEGDA) (Singh et al., 2022). Notably, ongoing research also explores scaffold-free, self-assembling approaches for bioengineering articular discs (Vapniarsky et al., 2018).

Various cell types can be used to seed these scaffolds, comprising stem cells, induced pluripotent stem cells (iPSCs), and differentiated chondrocytes (Acri et al., 2019; Zhao et al., 2024).

Growth factors prominently used in articular disc engineering include transforming growth factor-β1 (TGF-β1), fibroblast growth factor (FGF), and insulin-like growth factor (IGF) (Johns and Athanasiou, 2008; Kalpakci et al., 2011).

In an *in vitro* study, Yi et al. investigated 3D-printed polymer scaffolds coated with polydopamine (PDA) and combined with decellularized extracellular matrix (dECM) hydrogel for TMJ disc reconstruction, reporting promising outcomes in promoting chondrogenesis and fibrogenesis (Yi et al., 2021).

For mandibular condyle engineering, scaffolds can include the mentioned synthetic materials but also mineralized components like hydroxyapatite (HA) (Ciocca et al., 2013; Konopnicki and Troulis, 2015). While synthetic materials offer the advantages of an unlimited supply and straightforward manufacturing, mineralized scaffolds closely mimic the native bone structure, provide enhanced mechanical strength, and exhibit osteoconductive properties (Acri et al., 2019; Howlader et al., 2017). A combination of MSCs and growth factors, such as TGF-β1, FGF, and BMP-2, can be used to bioengineer the skeletal condyle (Konopnicki and Troulis, 2015; Grayson et al., 2010).

Engineering the glenoid fossa poses significant challenges due to its complex morphology and cartilage-bone interface. Notably, only few studies have addressed this aspect (Aryaei et al., 2016). A 2019 review by Acri et al. proposed combining MSCs with calcium phosphate, HA, or PCL scaffolds and BMP-2, SOX9, and VEGF for the skeletal part. For cartilage engineering, they recommended using MSCs with PLA, PCL, or alginate scaffolds and growth factors like BMP-2, TGF-β1, or IGF-1 (Acri et al., 2019).

A summary of materials for tissue engineering is presented in Table 3, providing an updated version of the work by Acri et al. (2019).

**TABLE 3:** Summary of materials for tissue engineering different parts of the TMJ.

		Scaffold		Cells	Additives
Articular discus		Natural	ECM sheets, fibrin, collagen, chitosan	Stem cells, iPSCs, differentiated chondrocytes	TGF-β1, FGF, IGF
		Synthetic	PLA, PCL, PGA, PLLA, PLGA, PEGDA		
Mandibular condylus		Mineralized	HA	MSCs	TGF-β1, FGF, BMP-2
		Synthetic	PLA, PCL, PGA, PLLA, PLGA, PEGDA		
Glenoid fossa	Skeletal part	Mineralized	calcium phosphate, HA	MSCs	BMP-2, SOX9, VEGF
		Synthetic	PCL		
	Cartilagous part	Natural	alginate	MSCs	BMP-2, TGF-β1, IGF-1
		Synthetic	PLA, PCL		

Abbreviations: ECM: extracellular matrix, PCL: polycaprolactone, PLA: polylactide, PGA: polyglycolic acid, PLLA: poly-L-lactic acid, PLGA: poly-L-lactic-co-glycolic acid, PEGDA: photopolymerized hydrogel polyethylene glycol diacrylate, HA: hydroxylapatite, iPSCs: induced pluripotent stem cells, MSCs: mesenchymal stem cells, TGF: transforming growth factor, FGF: fibroblast growth factor, IGF: insulin-like growth factor, BMP: bone morphogenetic protein, SOX: SRY (sex determining region Y-)box, VEGF: vascular endothelial growth factor.

Regenerative treatments using stem cells have demonstrated promising outcomes in the management of degenerative TMJ disorders, showing significant chondrogenic regenerative potential (De et al., 2019). This was exemplified in an animal study by Köhnke et al., where histological analysis revealed a substantial increase in cartilage thickness in stromal-cell-treated groups compared to untreated controls (Köhnke et al., 2021). These findings provide a valuable foundation for ongoing research in bioengineering and regenerative medicine. However, these regenerative approaches are primarily effective in early to moderate disease stages and require further validation in human trials (Matheus et al., 2022; Zhang et al., 2015).

### Recent advancements in 3D printing

Bioprinting represents a cutting-edge development in tissue engineering, advancing the scope of reconstructive surgery by enabling the production of intricate, multi-tissue structures with cellular viability intact. Rooted in the principles of three-dimensional printing, bioprinting integrates living cells, biomaterials, and bioactive compounds into precise, layer-by-layer arrangements to mimic native anatomical tissues (Ramadan and Zourob, 2021). The foundation of bioprinting lies in data derived from advanced imaging modalities such as CT and MRI, which are converted into CAD-models for custom tissue fabrication. Techniques such as inkjet, extrusion, laser-assisted bioprinting (LAB), and stereolithography utilize these models to create patient-specific grafts tailored to the complex structures of the TMJ (Bishop et al., 2017).

Other more sophisticated biofabrication techniques, permitting a fast processing in volume and permitting the use of hydrogel-precursors with undesirable rheological properties to be extruded, should be considered in the future to produce such kind of complex structures, including digital-light processing or volumetric 3D printing (Li et al., 2023; Rodríguez-Pombo et al., 2022).

Reconstruction of the TMJ is challenging due to the structural diversity and biomechanical demands. Bioprinting offers a promising approach, enabling precise customization of each TMJ component to address these complexities effectively. Particularly, bioprinting of fibrocartilaginous tissue for the TMJ holds promising future prospects, driven by ongoing research into cartilage bioprinting techniques, including scaffold-based, scaffold-free, and *in situ* bioprinting approaches (Wu et al., 2021). While a substantial body of literature exists on cartilage bioprinting in fields such as bioprinting of the meniscus, intervertebral disc, ear, and nose, research specifically focused on bioprinting TMJ fibrocartilaginous tissue remains limited (Hu et al., 2023; Perera et al., 2021).

Helgeland et al. explored the use of genipin-crosslinked 3D-printed gelatin scaffolds as a promising platform for regenerating TMJ cartilage. Their findings highlighted the ability of these bioprinted scaffolds to enhance the attachment, survival, and chondrogenic differentiation of human bone marrow-derived mesenchymal stem cells (hBMSCs). This demonstrates the scaffolds’ potential for supporting cartilage tissue engineering in TMJ repair and regeneration applications (Helgeland et al., 2021).

Advanced printing methods, such as integrated tissue and organ printing (ITOP), allow for the simultaneous deposition of multiple bioinks (i.e., a combination of hydrogels, cellular components, and bioactive additives) to create composite tissues (Gungor-Ozkerim et al., 2018; Park et al., 2022; Kang et al., 2016). Additionally, the use of 3D-printed surgical guides facilitates accurate graft placement during implantation, further enhancing clinical outcomes (Park et al., 2022).

**FIGURE 3:**
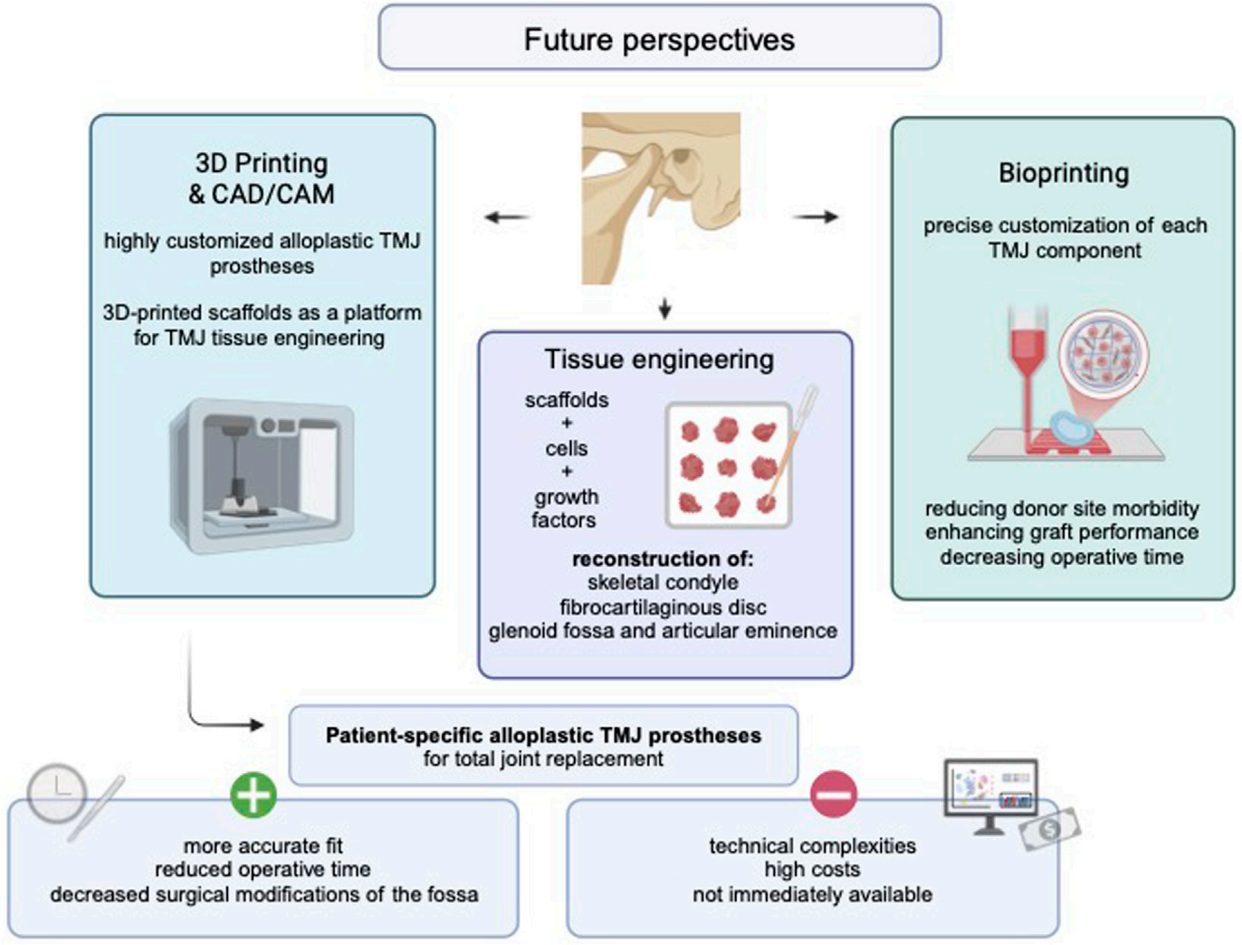
Graphical abstract presenting future perspectives in TMJ surgery. Created with BioRender (BioRender Inc., Toronto, Canada). Abbreviations: CAD/CAM. computer-aided design/computer-aided manufacturing, TMJ: temporomandibular joint.

Despite its potential, bioprinting for TMJ reconstruction remains in a translational phase, with limited clinical application and minimal progress in animal models. Currently, only a few bioprinted tissues, such as dermal, skeletal, cartilagous, or hepatical tissue, have been successfully transplanted into animals (Yang et al., 2021; Di Bella et al., 2018; Alawi et al., 2023). Ongoing research focuses on optimizing scaffold materials, improving bioink formulations, and understanding cellular behavior within engineered tissues. While animal models have demonstrated promising outcomes, significant challenges remain in achieving long-term stability, integration, and functionality of bioprinted TMJ components (Hu et al., 2023; Salah et al., 2020). As bone tissue engineering using LAB and cartilage bioprinting have been explored in isolated studies involving mice, small animal models, such as mice and further rabbits, represent a logical next step in the translational advancement of bioprinting (Keriquel et al., 2017; Apelgren et al., 2019). Future perspectives in biomaterials and manufacturing techniques are summarized in a graphical abstract, as presented in Figure 3.

### Tissue-engineered TMJ components in the growing patient

As a forward-looking approach, tissue-engineered TMJ components represent a promising biological alternative, particularly in pediatric patients. Several studies have demonstrated the successful implantation of tissue-engineered organs in growing children. For example, in 2012, Elliott et al. reported the implantation of a tissue-engineered trachea in a 12-year-old patient, which remained functional and demonstrated growth compatibility over a 2-year follow-up period (Elliott et al., 2012). Similarly, tissue-engineered vascular grafts have been successfully used in children with congenital heart disease, showing favorable safety profiles and long-term feasibility (Sugiura et al., 2018). Notably, children may benefit even more than adults from engineered tissues that integrate fully with the host organism and adapt to somatic growth. Growth-adaptive grafts are capable of accommodating the dynamic anatomical changes of the developing craniofacial skeleton and promoting host-mediated remodeling. Moreover, long-term durability is a critical consideration in pediatric patients due to their extended life expectancy and the cumulative mechanical demands placed on reconstructive materials (Ott, 2012). Although tissue-engineered TMJ components remain in the preclinical stage, their future clinical translation holds particular promise for growing patients.

### Approval pathways and ethical challenges

The FDA approval process for alloplastic TMJ devices follows the medical device regulatory pathway, typically requiring premarket approval (PMA) due to their classification as Class III devices, which involves rigorous evaluation of safety, efficacy, and long-term performance through clinical trials. Manufacturers must submit extensive data, including biocompatibility, mechanical testing, and clinical study outcomes (FDA, 2021). In contrast, tissue-engineered TMJ components fall under the category of combination products, involving both biologics and devices, and are regulated through a coordinated approach involving the Center for Devices and Radiological Health (CDRH) and the Center for Biologics Evaluation and Research (CBER) (NIH SEED, 2023). These constructs must undergo preclinical studies demonstrating functional integration, followed by Investigational New Drug (IND) applications and phased clinical trials before Biologics License Application (BLA) approval, making their translational pathway considerably more complex and time-intensive (Nordberg et al., 2022).

The use of alloplastic and tissue-engineered TMJ reconstruction in pediatric patients raises ethical concerns related to growth compatibility, long-term safety, and consent. Alloplastic devices may require multiple revision surgeries over a lifetime, while tissue-engineered constructs remain experimental with limited longitudinal data (Laventhal et al., 2012). Clinical studies involving children also face higher regulatory and ethical hurdles compared to adults, including stricter risk-benefit evaluations, assent requirements, and logistical challenges, which often result in fewer pediatric trials and delayed innovation (PREP, 2024). Key ethical issues include proxy decision-making, irreversible interventions, and the preservation of future treatment options.

## Discussion

Recent advances in digital simulation and additive manufacturing have catalyzed a paradigm shift in TMJ reconstruction. Customized alloplastic prostheses, enabled by VSP, have markedly improved both functional and aesthetic outcomes, while emerging regenerative strategies such as tissue engineering and bioprinting offer promising new directions. However, the benefits, limitations, and current challenges associated with these innovations must be critically evaluated.

The integration of VSP has significantly advanced TMJ reconstruction by shifting from conventional freehand techniques to precise, patient-specific approaches. Digital simulations and 3D-printed guides have streamlined operative procedures and enhanced the accuracy of graft shaping and prosthetic placement, thereby improving both functional and aesthetic outcomes (del Castillo Pardo de Vera et al., 2024; Mian et al., 2021; Dowgierd et al., 2021). Notably, the surgical approach in open TMJ procedures presents a significant challenge due to the close anatomical proximity to the branches of the facial nerve. To minimize the risk of injury to motor branches and the occurrence of neuropathic pain, thorough preoperative and intraoperative anatomical planning is essential (Esmaeelinejad and Sohrabi, 2015). Although various replacement systems are commercially available, ongoing innovations increasingly favor patient-specific devices. In clinical practice, maxillofacial surgeons must carefully balance the benefits and limitations of each system to determine the most appropriate intervention for individual patients.

While all four of the mentioned TMJ replacement systems (i.e., Zimmer Biomet, TMJ Concepts, KLS Martin, and Materialise) offer customized devices with similar components and materials, subtle differences persist (Materialise, 2023; KLS Martin Group, 2024; Giannakopoulos et al., 2012; Aagaard and Thygesen, 2014). Additionally, it is important to note that there is currently a paucity of published work on the systems developed by Materialise and KLS Martin.

Historically, autologous reconstruction was the mainstay for TMJ replacement. However, the significant complications and donor site morbidity associated with autologous grafts have driven the shift toward alloplastic prostheses, which generally exhibit lower complication and revision rates along with high survival outcomes (Wax et al., 2000; Sidebottom, 2013; Saeed et al., 2003; Amarista et al., 2020). Consequently, alloplastic TMJ reconstruction has been widely adopted despite certain contraindications, including its use in growing children and in patients undergoing adjuvant therapy for tumors (Pedersen et al., 2021; Borbon et al., 2023).

Recent advances in additive manufacturing have further refined these alloplastic TMJ replacement devices. For instance, approximately 27% of metal TMJ components are now produced using 3D printing, with this technology poised to dominate the future of patient-specific devices (Mercuri et al., 2022). Ongoing research is focused on developing increasingly individualized systems that better replicate native joint biomechanics and enhance functional outcomes. Nevertheless, challenges such as warping, deformation, and porosity, arising from heat transfer and rapid cooling during metal 3D printing, persist (Mercuri et al., 2022). Addressing these issues will require advanced methods, such as hot isostatic pressing and improved surface processing, to enhance durability and fracture resistance under masticatory forces (Leuders et al., 2015; Tammas-Williams et al., 2017). Despite challenges such as high costs, technical complexities, and the requirement for extensive and robust datasets, VSP and customized alloplastic TMJ reconstruction devices mark a paradigm shift in TMD management.

Innovative regenerative strategies have further broadened the scope of TMJ reconstruction. Tissue engineering offers a promising alternative to conventional options by leveraging advanced scaffolds, diverse cell types, and growth factors to replicate native anatomical structures and restore function (Acri et al., 2019). However, significant challenges remain in optimizing materials and methods for the TMJ’s complex components. Key hurdles include scaling up constructs in size and thickness, refining mechanical properties to enhance biomimetic performance, and thoroughly evaluating local and systemic biological responses to tissue-engineered components *in vivo* (Donahue et al., 2019). Moreover, new biomaterials, such as protein-based matrices derived from human blood or perinatal tissues, exhibit excellent biological responses and warrant consideration for TMJ reconstruction applications (Santos et al., 2018; Deus et al., 2020).

In this context, bioprinting naturally emerges as a complementary extension of tissue-engineered approaches, offering the capacity to precisely position cells, biomaterials, and bioactive compounds within three-dimensional constructs. The integration of bioprinting into TMJ reconstruction holds the potential to revolutionize maxillofacial surgery by reducing donor site morbidity, decreasing operative time, and enhancing graft performance (Salah et al., 2020). Considering the distinct structural organization and cellular composition of the TMJ, promising strategies include i) bottom-up tissue-engineered approaches, that permit to start with small scale elements that could be arranged hierarchically and with special control up to the macroscale, and ii) the combination of different biofabrication techniques to produce heterogeneous and complex structures, including the combination of extrusion and volumetric bioprinting, extrusion-based bioprinting, melt electrowriting, 3D-printing, and electrospinning (Gaspar et al., 2020; Ribezzi et al., 2023; Ainsworth et al., 2023; Ejiohuo, 2023). Despite their innovative potential, these regenerative strategies remain in the early stages of development, with most studies limited to *in vitro* experiments or small animal models. Although a few methods may eventually be integrated into clinical practice, many are likely to be abandoned as challenges related to scaling, mechanical stability, and biological integration are addressed. Consequently, real-world clinical outcome data on tissue engineering in TMJ reconstruction are currently unavailable. In contrast, population-based studies on alloplastic TMJ prostheses have demonstrated excellent survival rates (Amarista et al., 2020; Gerbino et al., 2024). Although evidence on long-term outcomes is still limited, several authors have reported favorable 10-year results for both patient-specific and stock prostheses with respect to functional improvement and pain reduction (Rajkumar and Sidebottom, 2022; Leandro et al., 2013). Implant survival at 10 years has been reported as 94.7% (Rajkumar and Sidebottom, 2022). To our knowledge, only one study has evaluated TMJ devices with a follow-up exceeding 20 years (Wolford et al., 2015).

TMJ reconstruction has seen transformative advancements through digital technologies such as VSP and additive manufacturing, enabling highly accurate, patient-specific alloplastic prostheses. However, three major challenges remain: the lack of growth adaptability in pediatric patients, material and mechanical limitations in 3D-printed implants, and the translational barriers facing regenerative strategies. Emerging technologies, such as 4D bioprinting, gene therapy, and bottom-up tissue engineering, offer promising solutions by enabling dynamic, biologically integrated constructs that can better replicate the complexity of the native joint. To realize these innovations clinically, interdisciplinary collaboration between engineers, surgeons, and biologists is essential, alongside rigorous preclinical testing and ethically sound clinical trials, especially in pediatric populations.
